# Pilot Testing of an Intensive Cooking Course for New Zealand Adolescents: The Create-Our-Own Kai Study

**DOI:** 10.3390/nu10050556

**Published:** 2018-04-30

**Authors:** Katherine Black, Carla Thomson, Themis Chryssidis, Rosie Finigan, Callum Hann, Rosalie Jackson, Caleb Robinson, Olivia Toldi, Paula Skidmore

**Affiliations:** 1 Department of Human Nutrition, University of Otago, P.O. Box 56, Dunedin 9054, New Zealand; katherine.black@otago.ac.nz (K.B.); carla.thomson@otago.ac.nz (C.T.); rosie.finigan@otago.ac.nz (R.F.); jacro835@student.otago.ac.nz (R.J.); caleb.robinson@otago.ac.nz (C.R.); tolol778@student.otago.ac.nz (O.T.); 2 Sprout Cooking School and Health Studio, 89 Sir Donald Bradman Drive, Hilton, South Australia 5033; themis@sprout.edu.au (T.C.); callum@sprout.edu.au (C.H.)

**Keywords:** adolescents, cooking, diet, self-efficacy

## Abstract

The role of cooking on health and wellbeing is a recent area of scientific interest. In order to investigate this role, a cooking program that is suitable for each target population is needed e.g., a program designed for American or Australian children might not be appropriate for teenagers in New Zealand. As there was no similar previously evaluated program already available, the study’s purpose was to test an intensive cooking intervention on cooking confidence and knowledge amongst a group of adolescents from Dunedin, New Zealand, and to assess its acceptability to participants. This five-day program comprised interactive cooking sessions and informal nutrition education and ran from 9 a.m. to 3:30 p.m., Monday to Friday during school holidays. Participants completed questionnaires on cooking skills and confidence at baseline and the end of intervention and took part in a group interview, which aimed to investigate the acceptability and outcome of the program. Twenty-one participants aged between 12 and 16 years old completed the program. At the end of the program, significant increases were seen in both skills and confidence levels, and feedback from the group interview indicated that the participants enjoyed the program and that it provided additional results other than those that were cooking related.

## 1. Introduction

Poor dietary intakes are believed to be one of the contributing risk factors for the current global burden of chronic disease [1]. Of particular concern is the rise in the number of convenience meals consumed and foods eaten away from home, as these could have detrimental effects on health. Convenience foods tend to be higher in total fat, saturated fat and consequently are more energy dense than those prepared at home [2]. Meals prepared at home tend to be higher in nutrients required for optimal health such as fibre, vitamins and minerals [3]. The importance of cooking at home has been shown in previous studies which report increased fruit, vegetable and fibre consumption and decreased fat and processed food intake amongst those who are more frequently involved in food and meal preparation, all of which are indicative of a healthier diet [3,4,5].

The importance of home cooking is now also appearing as a public health message. For example, it is highlighted in the USA in the American Strategy For Action component of the 2015–2020 Dietary Intakes Guidelines for Americans which states “Teach skills like gardening, cooking, meal planning, and label reading that help support healthy eating patterns” [6]. Previously, the teaching of cooking skills is something that was transmitted from generation to generation in the home or via formal education at school [7,8]. However, this is no longer the case and the majority of young people may be missing out on these life skills. In New Zealand, the Home Economics curriculum has undergone changes which has meant that rather than “teaching of life skills” (including cooking) students are now taught “informed consumer choices”, meaning many schools are no longer providing the cooking skills required to prepare meals from basic ingredients at home [9].

Although cooking at home can provide health benefits [3,4,5], there are several barriers to preparing healthy meals at home which include a lack of cooking skills, a lack of confidence to prepare meals at home, poor knowledge on how to prepare foods as well as a lack of time to prepare meals [10,11]. A particularly important barrier to behaviour change is the concept of confidence [12], commonly referred to as perceived self-efficacy. For adolescents, improving self-efficacy for cooking may not only help to motivate them to attempt to cook, but also help them to deal with stress, failures and overcome future barriers associated with cooking [13]. This is especially true given the lack of cooking opportunities available to them.

Addressing some of these barriers can be achieved by delivering hands-on cooking interventions. It is imperative that any intervention program is specifically designed for a target population and takes into account their dietary and cultural influences. Previous research in this area has shown that cooking interventions provide beneficial effects on food intake and health in adolescents [13,14,15,16,17,18,19,20,21,22]. However, the majority of published cooking interventions with adolescents have been conducted in the Northern hemisphere [13,14,15,16,17,18,19,20,21,22], where the price and availability of many foods, particularly seasonal fruit and vegetables, is likely to be very different to New Zealand. All of the interventions vary greatly in their cohort characteristics (ages, gender ratio, socio-economic status, ethnicity) and vary in duration from five days [17] to five months [19], and intensity, with some providing intensive full day programs over a week [17] whilst others delivered material in shorter blocks over several weeks [15,23].

At present there are no data on the efficacy of cooking interventions in New Zealand adolescents. Therefore, the current mixed methods pilot study was designed to test the effects of the Create Our Own Kai (COOK) study, where Kai is the Māori (New Zealand indigenous population) word for food. COOK is an intensive five-day cooking intervention on cooking confidence and knowledge amongst a group of adolescents and to assess its acceptability to participants.

## 2. Materials and Methods

### 2.1. Recruitment

We aimed to recruit 24 participants from school years 9–11 (participants ages 12–16 from Dunedin, New Zealand. Participants were recruited via social media, email, word of mouth, posters and via presentations at local school assemblies. Participants were required to have their own means of transport to and from the COOK study kitchens, but there were no other enrolment exclusions. Prior to participation both the participant and one of their caregivers provided written informed consent. Parents and children were required to provide written informed consent before entering the study. Ethical approval for the study was obtained from the University of Otago Human Ethics Committee.

### 2.2. Cooking Program

Participants were asked to attend a five-day cooking program which was conducted from 9:00 a.m. to 3:30 p.m. (approximately) Monday to Friday. Upon arrival at the study kitchens on Monday morning, participants were escorted to a room where they completed baseline questionnaires. Anthropometry measures were then taken in private, in a side room. Following this, the format of the cooking program was explained to the participants. Then participants were taken into the study kitchen and allocated into pairs who would cook and work together for the duration of the program.

Where possible, pairing was undertaken to avoid those who knew each other being paired together and to purposely pair those with the same food allergies, dislikes or food requirements based on religious beliefs. An allergen management plan was developed to minimise risk of cross-contamination of allergens. The cooking program was based on a commercial cooking program developed for use in schools in Adelaide, Australia (Sprout, Adelaide, Australia). The recipes had no added salt and were designed to be as healthy and nutritious as possible. Both sweet and savoury recipes were used, and desserts were made with as little added sugar as possible. Adaptations were made to these Australian recipes to incorporate (a) seasonal fruit and vegetables; (b) affordable ingredients easily available in Dunedin and (c) culturally appropriate practices. The recipes used in the program yielded two serves.

Examples of modifications to the program include being observant of Māori customs [24]. These cultural practices also align with good health and safety practice. Examples of this are that food should never be passed over the head. Fridges/freezers used to store food should be clearly marked and not used for any other purpose. Tables or other surfaces that are used for food should not be sat on and tea towels should only be used for the purpose of drying dishes and washed separately from all other soiled linen. The other major modifications were around the food and recipes provided. As New Zealand is a small country, with a population of five million, and it’s nearest neighbor, Australia, is over 2000 m away, the cost of food, particularly imported food is high. The price of fruit and vegetables fluctuates throughout the year, with the cost of these being four to five times higher when out of season. For example, a green capsicum (pepper) can cost $2 in summer and up to $12 in winter, the price of pumpkins can vary from $2 to $15, courgettes from $5 a kg to $16 a kg and the price of fruit when in season can be twice the price of that in Australia. Therefore, the recipes for the program were changed where necessary, or seasonal alternatives were given as part of the recipe. One of the original recipes was for a prawn curry, as prawns are expensive in New Zealand, and also to provide a vegetarian recipe, this was substituted with tofu for the intervention week. Participants were also given information on how to use chicken in the recipe instead. Another recipe was for chicken and asparagus couscous. Asparagus is cheap and freely available only during summer so information on substitutes such as frozen peas or pumpkin was given in the recipes so that these could be made all year round. Each recipe also contained tips on how to bulk up recipes so they could serve more people by either adding more ingredients, such as vegetables, or by making affordable side dishes such as homemade basic bread.

Each six-hour day consisted of watching a chef and a dietitian demonstrate food preparation (up to three recipes per day). In these demonstrations participants were shown how to cook the recipes and given other relevant information such as seasonal ingredient substitutes, brief nutrition information on ingredients, or the cheapest or best place to buy these ingredients. These demonstrations generally lasted around 15 min. Immediately after each demonstration, participants cooked the same meal in their allocated pairs before consuming the meal in one large group. For each recipe, study assistants pre-prepared trays of portioned raw ingredients for each participant pair. The trays included a two-serve recipe, with some items, e.g., spices and herbs, allocated more liberally so the students could adjust to their own taste preferences. Participants were also given four-serve versions of the recipe to take home. The cost of ingredients for each of these recipes was under $10 NZD.

Interspersed with the hands-on cooking were non-cooking talks and demonstrations. These activities included nutrition, hygiene, food safety, basic cooking skills (such as chopping) and cooking techniques, food preparation, seasonality of food and shopping local, recipe ideas and development, selecting ingredients, writing a meal plan and preparation list, meal budgeting as well as how to shop. Representatives from KiwiHarvest Dunedin, a food rescue program, covered topics of food waste, best before and use-by dates, re-using food and reducing the amount of food that enters landfill.

On the Monday, participants were asked to invite a family member to lunch on Friday. This meal was prepared by the participants. Therefore, time was set aside every day to allow participants to develop, adjust and finalise recipes for a two-course meal to be made by them on a budget of $25. These recipes were sourced, altered, planned and shopped for by the participants. Guidance was provided by the COOK Instructors on acceptability of nutrition, palatability, costs and cooking techniques that were not taught in class. Participants were not permitted to purchase any pre-packaged or processed food. Therefore, participants were required to hand-make pasta, pizza dough, bread and desserts from scratch (“raw” ingredients). Instructors encouraged the participants to extend their skills and to try new and interesting recipes. On the Thursday morning, participants and instructors went together to a local supermarket to purchase the required ingredients for the following day’s lunch. Each pair of participants were given a $25 grocery voucher to purchase the ingredients needed for the two- course meal and a range of cupboard staples were provided from an “open pantry”. These included flour, milk, herbs and spices. Participants were given two and a half hours of preparation and cooking time before their guests arrived for their first course to be served at 12:00 p.m. The second course was then served at 12:30 p.m. Following this, participants completed the post-study questionnaires and took part in a group discussion where students were asked to provide feedback on the week.

### 2.3. Questionnaire

A questionnaire on cooking skills and confidence was completed at the start and again upon completion of the cooking intervention.

Current cooking: Participants were asked “How often do you shop or help to shop for groceries?” with response choices of ‘never’, ‘sometimes’, ‘often’ or ‘always’. They were asked an open-ended question on “How many times a week do you help prepare food for your evening meal?” and “In a normal week, how often do you prepare and cook a meal from basic ingredients?” Responses ranged from ‘never’ to ‘daily’. Participants also completed the New Zealand Adolescent FFQ (NZA-FFQ) [25], which is a 72 item FFQ. This manuscript reports NZA-FFQ questions related to takeaway and fruit and vegetable consumption, namely “How often do you consume takeaways with (a) your family and (b) your friends and (c) if there are healthy takeaway choices available do you choose them?” and how many serves of fruit and vegetables do they eat daily. The data from these questions is provided only in order to characterise the study population at baseline. The NZA-FFQ was not repeated at the end of the cooking week as for this pilot study it was used only to provide background intervention on the study population. The NZA-FFQ designed to assess intake over a longer period than that of the intervention, and will be assessed seven weeks after the intervention in the planned future study.

Cooking Skills: The relevant 18 items from the Cooking with a Chef confidence questionnaire [26] was used for these measures. The questionnaire contains two sections, with section two asking “Can you perform the following activities” The skills covered were either mechanical or preparation relates. Mechanical skills are the singular technical skills of cooking and those assessed were knife skills, basic cooking techniques, steaming, sautéing, stir-frying, grilling, poaching, baking, roasting, stewing, boiling/simmering and microwaving. Preparation skills are those activities that could involve multiple technical skills and those assessed were cooking raw meat, poultry and fish, making sauces and gravy from raw ingredients, preparing fresh or frozen green vegetables, preparing root vegetables, preparing fruit, using herbs/spices). The questions used a response of “yes” or “no”. A response of “I don’t know what this is” could be selected for the mechanical questions only. An answer of “yes” corresponded to 1-point, and an answer of “no” or “I don’t know” corresponded to 0-points. Responses from all 18-questions were grouped together to give a final cooking skills score. The maximum score that could be achieved for this section was 18. Questions were also broken down into either mechanical skills (12 questions) or preparation skills (six questions), to give an overall score within these categories. A maximum score of 12 could be given for mechanical skills and a maximum of 6 for preparation skills.

Self-efficacy/confidence scores were also calculated from the Cooking with a Chef questionnaire [26]. For each question from section two described above, where participants were asked if they could do particular tasks, they were also asked to rate how confident they were that they were able to do each task. Each skill had responses on a 0–4 scale with 0 corresponding with “not at all confident” and 4 “being very confident”, meaning that the self-efficacy mechanical score had a potential total of 48 and the self-efficacy preparation score had a maximum possible score of 24. The questions from section one were also used to create confidence scores. This section contains four questions related to general cooking confidence—“Able to cook from basic ingredients”, “Following a simple recipe”, “Tasting foods that you have not eaten before” and “Cooking new foods from recipes”. Each question was ranked on a scale from “not confident at all” (score = 1) to “very confident” corresponding to a score of 7, meaning the total score for this scale was 28. A Total cooking skills self-efficacy score was calculated based on all of the self-efficacy questions meaning that it had a potential maximum score of 72.

Enjoyment: There were five questions related to the enjoyment related to cooking (“I really enjoy preparing food”, “I think it is fun to prepare food”, “I think preparing food is very interesting”, “I enjoy preparing food even if it takes a lot of time”, “I enjoy preparing food a lot”). These questions were developed for the current study and were pretested for understanding in a convenience sample of adolescents. Responses to these statements ranged from “strongly disagree” which was given a score of 0 to “Strongly agree” corresponding to a score of 4. This meant that the potential maximum total enjoyment score was 20.

Healthy eating: The questions used were from the validated HELENA study questionnaire [27]. There were two questions on the taste of fruit and vegetables: “fruit tastes good” and “vegetables taste good”. Responses to these statements were “fully disagree” (0 points) to “fully agree” (4 points). These two statements were combined into fruit and vegetable taste score with a maximum of 8 points. The opinions on healthy eating scale included “Do you think that you should eat a healthy diet” with potential responses, “definitely not”, “probably not”, “maybe”, “probably yes”, and “definitely yes”, which corresponded to scores 0, 1, 2,3, 4 respectively. “I like the taste of healthy food”, “I feel better eating healthily” had responses “Fully agree” (score = 4), “agree” (score = 3), “Don’t agree or disagree” (score = 2), “Disagree” (score = 1) and “fully disagree” (score = 0). The maximum potential score for “attitudes to healthy eating” was 12.

Also included in the questionnaire were some open-ended questions, where participants were asked what their favourite recipes were and why, if they had tried new foods and whether they liked them, what their favourite and least favourite parts of the week were. These questions were used to guide the group discussion, which took place after participants filled in their post-study questionnaires and the researchers identified topics to pursue from these questionnaires. The session began with the following questions “How do you think the week went?”, “Will you cook again in the next week?”, “How would you describe this week to a friend?”, “Why is learning to cook important?” Further discussion was based on answers to these questions and responses from the questionnaires. A researcher took detailed notes of the content of these interviews and the rest of the study team also recorded notes which were all used for analysis. Responses were coded by hand and common themes were identified from these. 

### 2.4. Anthropometry

Standing height was measured at baseline, in duplicate to the closest 0.1 cm using a Wedderburn portable stadiometer from Dunedin. If a 0.5 cm difference occurred between measurements a third standing height was taken. Participants were barefoot and heads in the Frankfurt plane position. Weight was recorded with participants wearing light clothing and a 0.5 kg adjustment for clothes on a bioimpedance scale (BC418, Tanita, Tokyo, Japan) with an accuracy of 0.1 kg. The same equipment was used throughout the study and study assistants received anthropometry training by Level 1 ISAK accredited persons to ensure standardisation.

### 2.5. Statistical Analysis

Data was checked for normality using the Shapiro-Wilk test and no departure from normality was found. As differences in scores between groups pre and post intervention were tested using paired t-tests. For the cooking skill section “Don’t know”, responses were grouped with “no” responses, except for sautéing (where there were nine “Don’t know” responses at baseline. There was a maximum of one “Don’t know” response for each question. *p* values (95% CI), for change scores are presented for the mean difference whereas pre and post intervention values for individual questions are mean (SD) unless otherwise stated.

## 3. Results

Of the 24 participants enrolled, 21 completed the full study (one dropped out due to other commitments, which meant they would have missed two days of the programme, one did not want to work with their assigned partner and one did not want to attend at 9 am every day). Demographic details of those who completed the study are shown in Table 1, the majority of participants were New Zealand European (*n* = 14, 66.7%). All participants lived at home during the school term, and they lived in households of between three and nine people.

### 3.1. Food Choice and Meal Preparation at Baseline

One person reported not eating any fruit, and ten (47.6%) reported that they consumed two servings of fruit per day. Only nine participants reported eating three servings of vegetables per day. Only two (9.5%) participants had takeaway with friends less than once a week, ten (47.6%) reported having it once a week with friends, and seven (33.3%) had takeaway with friends twice to four times a week and one (4.8%) had it 5–6 times a week with friends. One (4.8%) participant had takeaways less than once a week with their family, 16 (76.2%) had takeaway with the family once a week, three (14.3%) reported having takeaway with their family two to four times a week and one (4.8%) participant had takeaway with their family five to six times per week. 

Mean frequency for meal preparation was 1.2 ± 1.4 times a week, with seven (33.3%) participants reporting that they never helped prepare food for their evening meal. For those participants who reported that they did cook, they reported cooking for a mean of 3.9 ± 2.0 people, range 0–8 people. The mean number of times per week that participants stated they shopped for groceries was 1.3 ± 0.66. Four participants stated they “Do not cook at all”, three cooked convenience/ready-made meals; two put together ready-made ingredients to make a meal; with ten preparing dishes from basic ingredients. 

### 3.2. Cooking Skills, Confidence and Enjoyment

General cooking scores increased by 5.2 (SD 3.1), out of a possible maximum of 28, from pre to post intervention (*p* < 0.001). The mean mechanical skill scores for the Yes/No questions increased from 7.4 (SD 3.4) to 9.6 (SD 2.9) and the mean preparation skill scores for the Yes/No questions increased from 3.5 (SD 1.7) to 5.2 (SD 1.4). Results for the confidence scores showed that increases were seen for the majority of both the individual questions comprising the Mechanical and Preparation scores (Table 2). Confidence in Mechanical skills significantly increased from 22.7 (SD 7.8) to 32.6 (SD 6.8) (*p* < 0.001). There was also an increase in Preparation skills confidence from 11.2 (SD 4.6) to 18.2 (SD 4.5) (*p* < 0.001). There was no significant increase in cooking enjoyment of 2.5 (SD 2.7), out of a possible maximum score of 20 (*p* = 0.057).

### 3.3. Healthy Eating

There was a significant increase of 1.14 (SD 0.91) from pre to post intervention for the Fruit and vegetable taste score, *p* <0.001. There was also a significant increase for Attitudes to healthy eating score of mean difference 2.38 (SD 4.81), *p* <0.001. For “I feel better eating healthily” three (14.3%) participants decreased their level of agreement on this statement, and nine (42.9%) remained at the same level of agreement. For the statement “I should eat healthily” pre-intervention one (4.8%) fully disagreed, one disagreed (4.8%), four (19.0%) neither agreed or disagreed, agreed and fully agreed. Post intervention none fully disagreed nor disagreed, one did not agree or disagree, four agreed, and 16 fully agreed.

### 3.4. Group Discussion Feedback

Results from the group interview showed a wide range of benefits for the cooking intervention were experienced by participants, and that the feedback about the course was overwhelmingly positive. One student said that they would have liked to have been allowed to choose their partner for the week, when discussed further the majority of students thought that it was beneficial to be partnered with someone they didn’t know, because “It was great to meet new people and to develop new skills”, “I learnt lots of great skills, tried new foods and learnt to work with others”. Participants reported that the atmosphere in the group encouraged them to be more adventurous and to try other new foods that they thought they would not like, such as fish “salmon, I didn’t like it but I tried it!”, “..fish, it tasted really nice…”, “All the dishes were new to me and I liked almost all of them”, “herbs.. I enjoyed them”.

In terms of trying new foods, every participant tried at least one new food during the course of the week. The food that was tried for the first time for most participants was tofu. Feedback on these foods from the group suggested that they thought that they ”would not like the texture or the taste, but I was surprised that I did”. The tofu curry dish cooked during the week was a favourite of around half of the participants. Banana ice cream also a favourite, and the desserts were universally liked—“soufflé was amazing”, “parfait was really delicious’’, “The ice cream was a good way to eat healthy food in a dessert that tasted amazing”. With regards to fruit and vegetables, avocado was tried the most for the first time during the intervention, closely followed by fennel and courgette. Participants found that they were “trying new foods”, “buying a greater variety of fruit and veg” and had “many healthier snack alternatives to consider now”.

The participants all agreed that they had learnt new skills, which weren’t limited to cooking skills, with comments on their favourite part of the course including “deboning lamb for our dish on Friday”, “It was great to meet new people and to develop new skills”, “..learnt lots of great skills, tried new foods and learnt to work with others”, “I’m super inspired to cook healthy, nutritious food for my family! This has set me up for life!” “It taught you a lot of kitchen skills and makes you much more confident in the kitchen”. Eating food that they had cooked together, and the social benefits, was also a favourite part of the weeks, with participants commenting “I enjoyed getting to know different people and cooking the big meal for everyone to share. These things brought everyone together”. They also particularly enjoyed eating their creations because “because I prepared it myself’”.

Participating in the budgeting and shopping activities also provided new lessons for many including, “How to shop properly on a budget” and “Identifying new foods at the supermarket that they had never looked at before”. Many of the participants had already also cooked some of the meals at home for their family and enjoyed the challenge of doing this outside of the classes—“Making the soufflé as it tasted really nice and it was something that I thought I would not be able to make because it was too difficult”.

## 4. Discussion

It appears that this intensive hands on cooking program was effective at increasing short term cooking confidence and improving the liking of fruits and vegetables among New Zealand adolescents. The qualitative feedback from participants supports the responses from the questionnaires and the week was deemed to be fun and something that participants would recommend to others.

Participant’s fruit and vegetable intakes are similar to those reported in the most recent New Zealand Adult National Nutrition Survey (ANS08/09). According to the latest ANS, 50.9% of males and 61.5% of females aged 15–18 years meet the recommended three or more servings of vegetables each day, with 61.2 % of males and 65.0% of females aged 15–18 years eating the recommended two or more servings of fruit each day. In the present study at baseline, ~57% (12/21) met the vegetable and ~57% (12/21) met the current recommendations for fruit intake [28], suggesting that the overall fruit and vegetable intake of this age group could be improved. Cooking confidence has been shown to be a determinant of cooking or preparing foods at home and therefore is an important determinant of the overall diet [12], as those involved with meal preparation at home are more likely to ingest a diet high in fibre, vitamins and minerals, as well as lower in fat and saturated fat [3]. However, we must remember that the current study only assessed the confidence levels immediately upon completion of the intervention with regard to baseline. It does appear that the majority of the participants were already involved in some sort of cooking prior to the intervention with only two (9.5%) stating that they did no cooking at home although this was not always the evening meal as 33.3% stated they never helped prepare the evening meal. In the Project EAT (Eating Among Teens) survey on adolescents (aged 11–18 years old), ability and frequency of meal preparation and cooking over the previous week showed that the majority of participants in Project EAT helped to prepare a dinner meal (68.6%), which is similar to the 67.7% in the current study [3,4]. However, Project EAT reported a much lower proportion who assisted with grocery shopping as nearly half assisted in grocery shopping (49.8%) in Project EAT [29] compared to nearly everyone in the current study. This may reflect cultural differences between the two studies or the slightly younger participants in the current study which may mean that younger adolescents still accompany their parents to the supermarket whereas older adolescents have more independence.

The findings of the current research with regards to cooking confidence are in line with those previously published, which suggest that a fun, interactive, hands on cooking program has the ability to improve cooking confidence in adolescents [14,15,16,17,18,19,20,21,22,23]. For example, Condrasky et al. [18] conducted an intensive cooking intervention over five days focusing on food preparation skills, food safety practices and basic nutrition principles, making it similar to the COOK study. Similar questionnaires as in the present study were administered before and directly after completion of the five full days of the intervention and in line with the current study they showed increased confidence in cooking and food preparation skills [8]. In adults, Jamie Oliver’s “Ministry of Food” program also showed significant increases in cooking confidence from baseline to post intervention. They were also able to show that this confidence continued for six months post-intervention, suggesting that cooking programs can have longer term impact [30]. However, that study was in adults and future research should investigate whether similar findings occur in adolescents. Similarly, longitudinal research in adults [31] shows that having cooking skills in early adulthood is associated with better nutrition related outcomes 10 years later, so providing skills to adolescents has the potential to result in lifelong benefits. 

The feedback received at the end of the week suggests that the intervention week was fun and that the participants believed that they had learnt life skills, as well as cooking-specific skills. These are important because if something is perceived as being fun then the individual is more likely to proceed with these behaviours. Parents also stated that the children had cooked the recipes they had undertaken in class for the families at night. If this pattern of behaviour was to continue beyond the intervention week then it would not only be beneficial for the participants but could also have a positive influence on the dietary habits of their families and communities. Very few changes were suggested for the COOK program, however, the budget for the final meal could be moved to the first meal planning session. The low dropout rate for a study involving adolescents provides further evidence that the COOK project is acceptable to this group of New Zealand adolescents. This suggests that it is possible to run a program that is culturally- and age-appropriate in New Zealand with a budget of $5 or less per head per-meal, as well as being nutritionally appropriate.

Also, of importance is the liking of foods like fruits and vegetables. One of the main barriers to dietary change or to ingesting a diet high in fruit and vegetables is the belief/perception that these foods do not taste good. These data suggest that the way these foods were incorporated into the recipes that were cooked resulted in an increased “liking” of these foods. In adults there is a relative risk reduction for coronary heart disease of 7% with a one serve per day increase in vegetable consumption [32]. Therefore, removing potential barriers to fruit and vegetable consumption could have implications for public health. This could have positive effects on dietary intakes and long-term health, especially when it is considered that according to the latest Adult Nutrition Survey, 50.9% of males and 61.5% of females aged 15–18 years are meeting the recommended three or more servings of vegetables each day. Sixty-one point two percent of males and 65.0% of females aged 15–18 years are eating the recommended two or more servings of fruit each day. In the present study at baseline, ~ 57% (12/21) met the vegetable, and ~57% (12/21) met the current recommendations for fruit intake [33], suggesting that the overall fruit and vegetable intake of this age group could be improved. Due to the short period of this pilot study we were unable to determine the effects that this increased liking of fruit and vegetables has on actual intakes, however, this does provide us with information which can be used to determine protocols for future studies which should assess the impact of dietary preferences on dietary intakes over a longer period. 

There are some limitations to the current study, including the small sample size, which means that results cannot be generalizable to the general adolescent population in NZ and it is likely that we have a biased sample who were interested in cooking. The next stage of the project is to test this intervention in randomized controlled trial with a much larger sample, which should provide results from a more representative sample. The use of questionnaires may be considered a limitation, but the majority of the questionnaires used were previously validated, and where possible validated and assessed for reliability in a population of New Zealand adolescents [24] and were pretested before use in this study. The use of parametric tests for Likert scale data has previously thought to be controversial but it has been shown that this is an acceptable analytical method [34].

The baseline data in combination with the increase in confidence and the group feedback on the course suggests that despite the wide variety of cooking involvement by the participants at the start of the program, it was effective for all levels of cooking ability. This is important from a public health perspective as it means that such a program is applicable to most and does not need to be tailored to specific groups based on current cooking ability.

## 5. Conclusions

As food choice in adolescence has been shown to track into later life, it is important that educational programs which can change beliefs are undertaken in this age-group as it could have long-term health benefits. This pilot study shows that the COOK program has been well designed for its target audience, it is effective at increasing cooking confidence and modifying taste preferences as well as being fun and enjoyable for participants. Therefore, the results of this pilot study confirm that the COOK program could be an effective tool for larger future studies to assess the impact of cooking classes on dietary behaviours and health of adolescent New Zealanders.

## Figures and Tables

**Table 1:** Participant characteristics; mean ± SD (range).

	Male	Female	Total
N (%)	11(52.4)	10(47.6)	21
Age (years)	14.54 ± 1.13 (12–16)	14.38 ± 1.19 (13–16)	14.48 ± 1.15 (12–16)
Height (cm)	170.31 ± 12.45 (146–190)	165 ± 7.80 (149–172)	168.29 ± 11.28 (146–190)
Weight (kg)	63.40 ± 15.89 (39.50–90.10)	56.64 ± 12.01 (44.90–77.60)	60.82 ± 14.76 (39.50–90.10)
Body Fat (%)	15.57 ± 9.05 (8.60–40.90)	27.96 ± 8.40 (18.70–39.60)	20.29 ± 10.85 (8.60–40.90)
BMI z-score N (%)			
Underweight	0 (0.0)	1 (12.5)	1 (4.8)
Healthy	8 (72.7)	6 (60.0)	14 (66.7)
Overweight	2 (18.1)	3 (30.0)	5 (23.8)
Obese	1 (7.7)	0 (0.0)	1 (4.8)
Ethnicity N (%)			
NZEO ^a^	5 (45.5)	9 (90.0)	14 (66.7)
Maori	2 (18.2)	0 (0.0)	2 (9.5)
Chinese	2 (18.2)	0 (0.0)	2 (9.5)
Middle Eastern	1 (9.1)	1 (10.0)	2 (9.5)
American	1 (9.1)	0(0.0)	1 (4.8)

^a^ NZEO: New Zealand European and other European.

**Table 2:** Mean (SD) scores for the individual cooking confidence questions and the mechanical and preparation scores.

	Pre	Post	
	Mean ± SD	Mean ± SD	*p*-Value *
Use knife skills	2.29 ± 1.10	3.24 ± 0.77	0.002
Basic cooking techniques	2.81 ± 1.08	3.38 ± 0.74	0.052
Steaming	1.24 ±1.00	2.48 ± 0.98	<0.001
Sautéing	0.67 ± 0.97	1.81 ± 1.33	0.003
Stir-frying	1.71 ± 1.06	2.70 ± 0.92	0.003
Grilling	1.33 ± 0.86	2.57 ± 0.93	<0.001
Poaching	1.38 ± 1.16	2.05 ± 0.86	0.041
Baking	2.52 ± 1.29	3.10 ± 0.94	0.109
Roasting	1.86 ± 1.06	2.76± 0.83	0.004
Stewing	1.38 ± 0.86	2.29 ± 0.85	0.001
Boiling/simmering	2.62 ± 1.02	3.00 ± 0.89	0.206
Microwaving	3.05 ± 0.97	3.38 ± 0.97	0.274
Mechanical Score	22.68 ± 7.81	32.62 ± 6.78	<0.001
Cook meat	1.75 ± 1.12	3.10 ± 0.94	<0.001
Make sauces	0.90 ± 0.77	2.62 ± 0.97	<0.001
Prepare green vegetables	2.57 ± 1.03	3.29 ± 0.72	0.013
Prepare root vegetables	2.19 ± 0.98	3.29 ± 0.78	<0.001
Prepare fruit	2.00 ± 1.30	3.10 ± 1.04	0.005
Use herbs/spices	1.86 ± 1.20	2.81 ± 1.17	0.013
Preparation score	11.19 ± 4.55	18.19 ± 4.48	<0.001
Total score	34.05 ± 12.00	50.81 ± 10.24	<0.001

* *p*-value: difference between pre and post intervention. Scores for each individual question ranged from 0 to 4. The maximum score obtainable from the Mechanical score is 48, the Preparation score 24 and the Total score 72.
